# Age-and sex-adjustment and the COVID-19 pandemic – transformative example from Italy

**DOI:** 10.1093/ije/dyaa139

**Published:** 2020-08-23

**Authors:** Valentina Gallo, Paolo Chiodini, Dario Bruzzese, Raj Bhopal

**Affiliations:** 1 Campus Fryslân, University of Groningen, Leeuwarden, The Netherlands; 2 Institute of Population Health Science, Queen Mary University of London, London, UK; 3 Department of Medical Statistics, London School of Hygiene and Tropical Medicine, London, UK; 4 Medical Statistics, University of Campania “L. Vanvitelli”, Naples, Italy; 5 Medical Statistics, University of Naples “Federico II”, Naples, Italy; 6 Usher Institute, University of Edinburgh, Edinburgh, UK

The COVID-19 pandemic is causing hundreds of thousands of deaths worldwide.^1^ Monitoring the pandemic to compare countries and regions is of paramount importance to understand the infection dynamics and to prepare health care systems to face its consequences. To date, it has been impossible to compare data coming from different countries and regions partly because of a failure to apply basic epidemiological principles (e.g. adjustment for age), with emphasis on the numbers of cases.^2^ Interpreting numbers of cases (and the rates derived from them, e.g. case-fatality ratio) is problematic given that these are heavily dependent on variable policies about testing for COVID-19 at population level, leading to potential underreporting, especially of people showing few or no symptoms. Mortality, on the other hand, does not suffer from difference in testing and case finding; however it is potentially subject to misclassification too, whenever its definition differs from that recommended by WHO: deaths for which the immediate or underlying cause of death can be reasonably ascribed to COVID-19.^3^ China^4^ first reported that mortality from COVID19 is strongly associated with age and steeply increases with age, with higher rates in males than females. Therefore, not adjusting for age and sex undermines meaningful comparison between populations, especially when the age structure of populations differs markedly, such as for comparisons between low- and middle-income countries with high-income countries.

To illustrate the importance of this principle, data on age and sex distribution of the first 4993 COVID-19 deaths in Italy, recorded until 23 March 2020,^5^ were used to calculate age- and sex-standardized figures in each Italian region. Assuming that the age- and sex-mortality rates remain constant over time, each data point can be interpreted as a standardized mortality trend ratio (SMTR), i.e. the ratio between observed deaths in a region on a specific day, over the expected deaths if that region had the same mortality as the Italian average on 23 March 2020 x 100. In Figure 1, the cumulative number of deaths by region is reported in panel A, and the daily SMTRs calculated on the cumulative deaths relative to the same period are reported in panel B.

**Figure 1:**
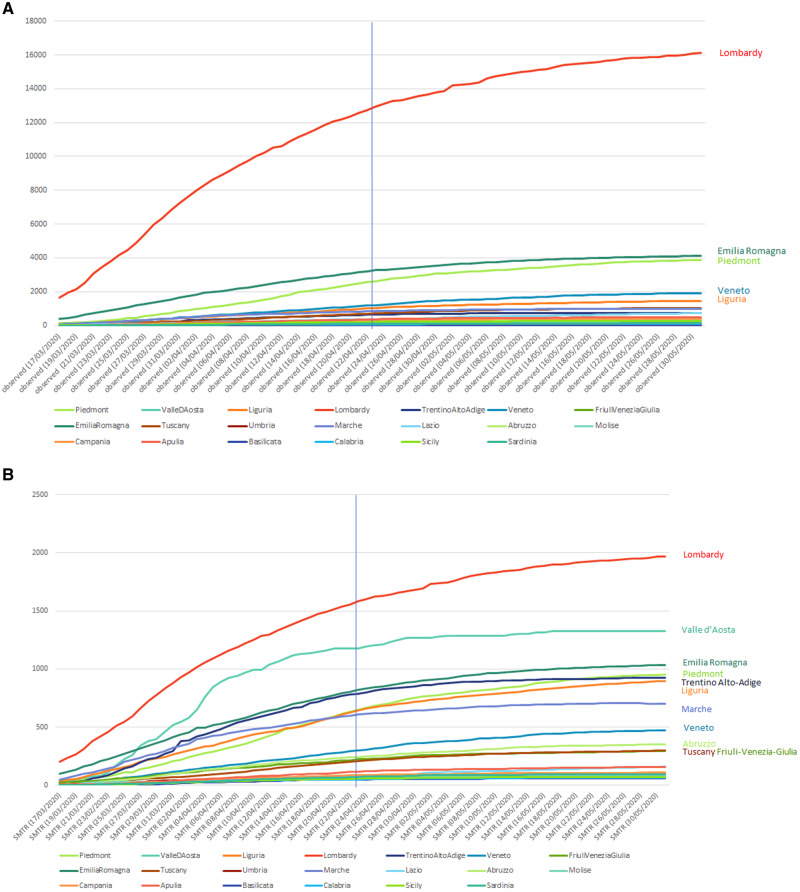
Absolute number of deaths by region (panel A) and Standardized Mortality Trend Ratios (SMTR) comparing observed vs. expected cases based on the age and sex distribution of the first 5019 Italian cases on 23 March 2020, and the age and sex structure (panel B) by region from 17 March to 3 May 2020 (vertical full line: 23 March 2020, the data for which were used for standardization)

Lombardy is the region rhat experienced the highest death toll by far, reaching 16 112 death by the end of May 2020. Emilia Romagna and Piedmont reached only about 4000 deaths (4114 and 3864, respectively), followed by the other regions all below 2000 deaths. However, once the underlying age- and sex-structure of the population was accounted for, the picture changed. Lombardy remained the region experiencing the greatest excess mortality with SMTRs almost 20 times higher (SMTR = 1968) than the national average (on 23 March); Valle d’Aosta (SMTR = 1323) was the second region for mortality followed by Emilia Romagna (SMTR = 1034) and Trentino-Alto Adige (SMTR = 924).

At the beginning of April, Marche experienced almost double the SMTR compared with Piedmont, but by the beginning of May the relative mortality between the two regions reversed. Veneto, among the morthern regions, was comparatively less affected with the SMTR only four times higher than the national average. Also, it emerged more clearly which regions were most successful in ‘flattening the curve’ (e.g. Valle D’Aosta, Trentino-Alto Adige, Marche) as opposed to those regions which were still experiencing COVID-19-related mortality, although at a lower rate of increase (e.g. Lombardy, Piedmont, Liguria).

Age- and sex-standardization is essential for monitoring the pandemic in space and over time. Our method has the limitations of assuming that the age- and sex-specific mortality rates remain constant over time (which we are addressing in ongoing work), and that COVID-19 related mortality is coded consistently across regions. However, if every country provided the WHO with the simple data required for calculating the SMTRs, the monitoring of trends across regions and over time would convey realistic approximations with minimal requirement for data, once age- and sex-specific COVID-19 mortality data from adequately representative populations were available.

## Conflict of interest

None declared.
